# Prebiotics and Probiotics: Healthy Biotools for Molecular Integrative and Modulation Approaches

**DOI:** 10.3390/ijms24087559

**Published:** 2023-04-20

**Authors:** Margarita Aguilera, Abdelali Daddaoua

**Affiliations:** 1 Department of Microbiología, Pharmacy School, University of Granada, 18071 Granada, Spain; maguiler@ugr.es; 2 Instituto de Investigación Biosanitaria (IBS), 18014 Granada, Spain; 3 Institute of Nutrition and Food Technology “José Mataix”, Center of Biomedical Research, University of Granada, Avda. del Conocimiento s/n. Armilla, 18016 Granada, Spain; 4 Department of Biochemistry and Molecular Biology II, Pharmacy School, University of Granada, 18071 Granada, Spain

The scope of this Special Issue is to highlight and expand our knowledge on the molecular mechanisms of prebiotics and probiotics, as well as to offer a broad overview of current advancements and future directions in this research field. In fact, these new biotools have become the main instruments to intervene and optimize several physiological functions of the microbiota. In this context, considerable efforts have been made to influence the intestinal microbiota through dietary complementary means in such a way that the health of the host should be positively and effectively affected.

The key role of beneficial bacteria in the intestine in promoting overall human health is generally accepted. Specifically, beneficial bacteria in the colon show many important functions, including increasing mineral homeostasis, strengthening the intestinal barrier, and regulating the immune response, and, therefore, positively affect human health and wellbeing. Probiotics and prebiotics are considered food supplements or bioproducts that exert key functions in maintaining human gut eubiosis; indeed, they are specially designed for this purpose. Furthermore, although it is often stated that functional foods are foods that provide benefits beyond their traditional nutritional value, by either enhancing a function of the body or reducing the risk of a disease, it is necessary to demonstrate their underlying molecular mechanisms.

Therefore, the current Special Issue covers an interesting collection of reviews and original research articles focusing on new biotools for integrative approaches dealing with prebiotics and probiotics. The selected studies examined a wide range of methodologies for discovering and implementing metabolic-based approaches, together with engineering and editing genomes, to enhance the beneficial properties of gut microorganisms.

The study performed by Martin Garcia-Gonzalez et al. [1], demonstrated that a mixture of honey hetero-glucooligosaccharides (hetero-GlcOS) synthesized from sucrose using immobilized *Escherichia coli* cells expressing the α-glucosidase enzyme, Mr-αGlu, displayed a higher residual activity and confirmed that isomelezitose is a potential novel prebiotic that could be included in healthier foodstuffs designed for human gastrointestinal balance maintenance. Similarly, Sara Deleu et al. [2], suggested that probiotics that can locally produce a high acetate concentration might be the way forward to deal with inflammatory bowel disease (IBD) management due to their beneficial effects on barrier-defected and inflammatory diseases. 

Huanghuang Dai et al. [3], found a positive correlation between the addition of glucosylceramide and the induction of changes in the metabolism of *Blautia coccoides,* which leads to an increase in the number of Gram-positive bacteria in an intestine-simulating environment by endowing tolerance to deoxycholic acid. At the same time, the findings reported by Notararigo et al. [4], clearly confirmed the immunomodulation of O-2-substituted-(1-3)-β-D-glucans in an in vitro model of intestinal gut mucosa after the stimulation of PMA-THP-1 cells with *E. coli* LPS, which triggered cellular response mechanisms. Hence, O-2-substituted-(1-3)-β-D-glucans is able to ameliorate immune system response in healthy individuals, as well as in individuals with some chronic inflammatory states. This finding is supported by Aleksandra Maria Kocot et al. [5], who summarized the role of biotics in the maintenance of intestinal barrier function and the need to understand the mechanisms by which biotics regulate this function. Likewise, Francisco Javier Ruiz-Ojeda et al. [6], posited that consumption of the new INN formula under investigation could improve the composition of the gut microbiota, thereby promoting a healthier gut microbiota more similar to that of an infant receiving exclusively human milk. 

It is clear that evaluating the effects of probiotics against pathogens, such as by comparing both viable strains and their metabolic products and using co-cultures of pathogens, provides insights into the influences they can exert and whether a specific probiotic can be used in case of infection. Yan Zeng et al. [7], confirmed that *Lactiplantibacillus plantarum* 14917 in dental caries presented an inhibitory impact on virulence expression genes and growth of *Streptococcus mutans* and *Candida albicans*, resulting in a reduced biofilm structure. Patrizia Malfa et al. [8], suggested that *Lactiplantibacillus plantarum* PBS067, *Lactiplantibacillus rhamnosus* LRH020, *Bifidobacterium animalis* subsp. Lactis BL050, and their combination, SynBalance^®^ Femme, are able to compete and prevent the development of the most common urogenital pathogens through several mechanisms, such as production of antimicrobial substances, competence in epithelial cell adhesion, and co-aggregation capacity. Likewise, Paola Zanetta et al. [9], obtained through screening key information related to the efficacy of viable probiotic strains of *Lactobacillus* CFS selected as the most efficient strain in the containment of oral pathogens.

Finally, it should be noted that in relation to next-generation probiotics (NGP), Alfonso Torres-Sánchez et al. [10], highlighted the importance of integrative techniques of multi-omics data analysis, such as machine learning and artificial intelligence, to characterize the effects of probiotics, including metagenomic, metatranscriptomic, and metabolomic technologies, with the aim of identifying losses of function of the microbiome associated with changes in health status. 

We hope that this Special Issue can further expand key knowledge on molecular mechanisms of new and improved prebiotics and probiotics, as well as providing a complete and comprehensive overview of current advancements in this area, and its future global projection and transferability to different key actors.
